# Serum-derived miR-574-5p-containing exosomes contribute to liver fibrosis by activating hepatic stellate cells

**DOI:** 10.1007/s11033-021-07008-2

**Published:** 2021-11-29

**Authors:** Xia Zhou, Ziyu Liang, Shanyu Qin, Xianxian Ruan, Haixing Jiang

**Affiliations:** 1 Department of Gastroenterology, The First Affiliated Hospital of Guangxi Medical University, Nanning, Guangxi China; 2 People’s Hospital of Guizhou Province, Guiyang, Guizhou China

**Keywords:** Liver fibrosis, Serum exosome, Hepatic stellate cell, miR-574-5p

## Abstract

**Aim:**

To investigate the association of serum exosomes miR-574-5p with liver fibrosis, and explore the effect and mechanism of serum exosomes on HSC activation.

**Materials and methods:**

Using serum samples collected from healthy adults and patients with liver cirrhosis, we extracted human serum exosomes via ultra-high-speed centrifugation, and co-cultured them with hepatic stellate cells (HSCs) line LX2. LX-2-mediated intake of human serum exosomes was examined by confocal microscopy. To induce liver fibrosis, we administered 20% CCl_4_ to mice intraperitoneally and adopted an exoEasy MIDI kit to extract serum exosomes.Liver fibrosis-related molecules were determined via qRT-PCR, Western blot, Masson staining, and Immunohistochemical staining.

**Results:**

Significantly high miR-574-5p levels were expressed in serum exosomes and were positively correlated with the expression of miR-574-5p, collagen deposition, and α-SMA expression in liver tissues of mice during liver fibrosis. Compared to healthy subjects, serum exosomes from cirrhosis patients were associated with higher expression of miR-574-5p. MiR-574-5p mimic promoted the expression of α-SMA and COL1A1 mRNA and protein in LX-2, whereas miR-574-5p inhibitor exerted no effect.

**Conclusion:**

This article demonstrates that miR-574-5p expression in serum exosomes is positively correlated with collagen deposition and HSC activation in liver tissues during liver fibrosis.Serum exosomes potentially activate HSC through the transfer of miR-574-5p to HSC during liver fibrosis.

## Introduction

Liver fibrosis occurs due to chronic liver damage and is attributed to multiple predisposing factors, including alcohol consumption, non-alcoholic steatohepatitis(NASH), viral hepatitis, autoimmune hepatitis, non-alcoholic fatty liver disease (NAFLD), and cholestatic liver disease [1, 2]. Hepatic stellate cell (HSCs) activation is the key event in hepatic fibrosis [3]. Factors, including toxins, hepatitis, steatohepatitis, and autoimmune diseases may trigger the activation of HSCs and transformation into myofibroblasts. Subsequently, the synthesis of extracellular matrix (ECM) promotes the progression of liver fibrosis [4, 5]. Therefore, the regulation of HSCs activation, proliferation, and apoptosis is crucial in the management of liver fibrosis [6].

Exosomes are small vesicles (about 30–150 nm in diameter) secreted by various cells and widely localized in plasma, saliva, urine, and other body fluids [7, 8]. They comprise proteins, lipids, nucleic acids, and other bioactive substances, which influence the progression of diseases via intercellular material and information transduction, or direct action on target cells [9, 10]. miRNAs enriched exosomes are delivered to HSCs [11], which is a crucial event in liver fibrosis. Serum exosomes can be applied in liquid biopsy for noninvasive detection and staging of liver diseases [12, 13]. Recent reports show that normal serum exosomes exhibit anti-fibrotic properties, and contain miRNAs with therapeutic effects against HSCs activation [14].

miRNA, a class of non-coding single-stranded RNA molecules about 22 nucleotides long is encoded by endogenous genes. It regulates post-transcriptional gene expression, which is an important mechanism for the development of many diseases [15]. Numerous studies have elucidated the mechanisms of miRNA in liver fibrosis [16, 17]. In a previous study, high miR-21 expression in fibrotic liver promoted HSC activation [18, 19]. Simultaneously, miR-29a inhibited collagen synthesis but was lowly expressed when HSC is activated [20]. miR-19b also exerts anti-fibrotic effects in the regulation of the transfroming growth factor-β(TGF-β) signaling pathway in HSC [21]. Jeongeun Hyun adopted a miRNA array to analyze the difference of miRNA expression in the process of liver fibrosis in mice and found that miR-574-5p increased most significantly in the fibrosis liver tissue [22]. However, the precise role of miR-574-5p remains unclear. Herein, we analyzed the expression of miR-574-5p in the serum exosomes during liver fibrosis and explored its possible mechanism of action in the process of liver fibrosis.

## Material and methods

### CCl_4_-induced hepatic fibrosis in mice

Male *C57BL/6* mice (7–8 weeks old) were provided by the experimental animal center of Guangxi Medical University, which we used to develop the liver fibrosis model. To induce liver fibrosis, the mice were intraperitoneally injected with 20% CCl_4_, 0.4 mL/100 g twice a week. Simultaneously, the control group was administered with the same dose of olive oil. Liver tissue and peripheral blood were collected at 2, 4, 6, and 8 weeks. The perfused liver tissues were fixed with 4% formalin or preserved at—8 °C. We subjected the fixed liver tissues to H&E staining, and then detected collagen via Masson staining. The expression of α-SMA was detected through immunohistochemistry analysis. Serum was separated from peripheral blood for further exosome extraction.Animal ethics review follows the Guiding Opinions on the Treatment of Laboratory Animals issued by the Ministry of Science and Technology of the People’s Republic of China and the Laboratory Animal-Guideline for Ethical Review of Animal Welfare issued by the National Standard GB/T35892-2018 of the People’s Republic of China.

### Collection of human serum

Human serum samples were collected from 12 patients diagnosed with hepatitis B virus F3/4 fibrosis and 10 healthy volunteers aged between 45 and 65 years. We excluded the effects of taking drugs and drinking within 24 h. No other potential diseases were found in all participants. The isolated serum samples were kept at −80 °C for further analyses. The study was approved by the ethics committee of the First Affiliated Hospital of Guangxi Medical University. All subjects signed informed consent.

### Collection of mouse serum

We collected the peripheral blood from *C57BL/6* mice in the control group and liver fibrosis group via a retro-orbital bleed. Blood samples were centrifuged at 3,000 rpm for 10 min at 4 °C to remove blood cells. The supernatant was then centrifuged at 16,000 g for 10 min at 4 °C to completely remove the larger vesicles. The serum samples were stored at −80 °C

### Serum EV purification

Human serum samples were centrifuged at low speed (300 g × 15 min 4 °C; 2000 g × 10 min 4 °C) and 10,000 g for 30 min to remove large vesicles. A W41Ti rotor (Beckman Coulter, Brea, CA, USA) was applied to centrifuge the supernatant at 100,000 × g for 90 min The pellets were washed in phosphate-buffered saline (PBS) to remove protein contaminants and centrifuged again at 100,000 × g for 90 min. The exosomes were resuspended in PBS. 8μgml of serum exosomes from normal adults and cirrhotic patients were co-cultured with LX-2 for 24 h respectively, Total RNA from serum exosomes and LX-2 were extracted by miRNeasy Kits (Qiagen) and NucleoZOL(GeneBank). We applied the exoEeasy Midi Kit (Qiagen) to isolate exosomes of mouse serum according to the manufacturer’s instructions. Briefly, 1 volume of buffer was added to 1 volume of the sample. The sample/Buffer XBP was mixed onto the exoEasy spin column and the device spun at 500 g for 1 min. We did another spin at 5000 g for 1 min to eliminate all the liquid. This was followed by the addition of 3.5 ml Buffer XWP and centrifugation at 3000 g for 5 min to remove residual buffer. Exactly 400ul buffer XE was added to elute the exosomes for electron microscopic examination. To extract serum exosomal RNA, 700ul Qiazol was added. The serum exosomes were examined via transmission electron microscopy (TEM). Nanoparticle tracking analysis (NTA) of exosomes was performed using zetaview PMX 110.

### Histology

Liver tissue was fixed with formalin, treated by conventional histology, embedded in paraffin, and sectioned (5 µm). Sections were stained with H&E staining (Solarbio, Beijing, China) and Masson trichromatic staining reagent (Bestbio, Shanghai, China) according to standard procedures to assess histopathological severity and collagen deposition in liver tissue. Photographs were captured with a light microscope (Olympus, Tokyo, Japan). Three fields in the section were randomly selected. Collagen was stained blue by Masson staining, Image J 6.0 software was used to analyze and calculate the collagen deposition area.

### Immunohistochemistry

Cut paraffin blocks into 5 μm thick slices. The following steps were implemented: routinely dewaxed,dehydration, the antigen was repaired in EDTA buffer for 3 min at high temperature and high pressure, then washed at 25 °C, incubated with 3% hydrogen peroxide for 30 min, washed, and incubated with 5% goat serum at room temperature for 15 min.Then, the sections were incubated with anti-α-SMA (1:250, ab124964, abcam) overnight at 4 °C, incubated with goat anti-rabbit IgG polymers (Zhongshan, Beijing, China) and horseradish enzyme for 30 min in turn at room temperature. Then, counterstained the slices with hematoxylin. The brown granules showing positive expression of α-SMA were observed in liver tissue. At least three magnification fields within the sections were randomly selected with an light microscope (Olympus, Tokyo, Japan), and α-SMA positive areas were calculated with ImageJ 6.0 software.

### HSC cultures and transfection

LX-2 cells were cultured in DMEM/10% FBS, seeded in 6-well plates, and then cultured in a serum-free medium for the next 24 h. LX-2 cells were treated with serum exosomes of healthy adults and cirrhotic patients for 24 h, respectively. Expressions of collagen α1(I) (COL1A1), α-SMA mRNA, and miR-574-5p were determined via qRT-PCR. miRNA mimic and miRNA inhibitor were transfected using Lipofectamine™ RNAiMAX Transfection Reagent (Invitrogen). To detect the expression of collagen α1(I) and α-SMA, we extracted RNA and protein at 24 h and 48 h, respectively. The miRNA-574-5p mimic, nc mimic, miRNA-574-5p inhibitor, and nc inhibitor were purchased from Jima.

### EV binding assays

The serum exosomes labeled with PKH-67 (20 µg/ml) were added to LX-2 and incubated at 37 °C for 12–24 h in the dark. The cells were washed with PBS and then fixed. We stained the nucleus with DAPI and examined it for fluorescence using a confocal microscope.

### RNA extraction and RT-qPCR

Total RNA from cultured HSC and liver tissue were extracted by nuclearol (GeneBank) and reverse-transcribed using PrimeScript™ RT Master Mix(TAKARA) following the manufacturer’s protocols. Quantitative real-time RT-PCR was performed using TB Green Premix Ex TaqTM II (TAKARA) with primers for α-SMA, collagen α1(I). Total RNA from serum exosomes and liver tissues were extracted by miRNeasy Micro Kit(Qiagen) and nuclearol (GeneBank) respectively, and then reverse-transcribed using miRcute Plus miRNA First-Strand cDNA Kit (TIANGEN). Quantitative real-time RT-PCR was performed using miRcute Plus miRNA qPCR Kit (TIANGEN). GAPDH (for mRNA) and U6 (for miRNA) acted as the reference gene (Table 1).

**Table 1:** Primer sequence of RT-PCR

	Forward	Reverse
α-SMA	AAAAGACAGCTACGTGGGTGA	GCCATGTTCTATCGGGTACTT
COL1A1	GAACGCGTGTCATCCCTTGT	GAACGAGGTAGTCTTTCAGCAACA
GAPDH	TGCACCACCAACTGCTTAGC	GGCATGGACTGTGGTCATGAG
U6	CTCGCTTCGGCAGCACA	AACGCTTCACGAATTTGCGT
Mouse miR-574-5p	CD202-0043(TIANGEN)	
Human miR-574-5p	CD201-0044(TIANGEN)	

### Western blotting

Protein was isolated through lysis with radioimmunoprecipitation assay (RIPA) buffer supplemented with 1:100 protease inhibitor (Solarbio, Beijing, China). The BCA protein assay kit (BOSTER, Wuhan, China) was used for protein quantification. Samples were separated by SDS-PAGE gels and transferred onto a PVDF membrane (Millipore). The membranes were blocked for 1 h with 5% skimmed milk and incubated overnight at 4 °C with primary antibodies: anti-CD9 (1:1000, ab92726, System Biosciences), anti-TSG101(1:2000, ab125011, System Bioscienc-es),anti-COL1A1(1:2000, ab260043, abcam), anti-α-SMA (1:5000, ab124964, abcam), GAPDH (1:1000, 41,549, CST). Then, after 3 times wash, the membranes were incubated with the HRP-conjugated secondary anti-Rabbit antibody for 1 h at room temperature. Immunoreactive bands were visualized using Immobilon Western HRP (GeneBank; USA) and analyzed in Image J software.

### Statistical analysis

Masson staining, immunohistochemical staining, and Western-blot results were analyzed by Image J 6.0 software. The expression levels of miRNAs in exosomes and liver tissues were determined by 2^−△△CT^. Data processing and statistical analyses were conducted using SPSS 20.0 software. Data were expressed as mean ± standard deviation. The software GraphPad 8.0 was used for plotting. Comparison of means between the multiple groups was achieved using One-way ANOVA, whereas an independent sample t-test was used for the comparison between the two groups. Spearman correlation coefficient was used for correlation analysis. *P* < 0.05 denoted statistically significant difference. Each experiment was conducted in three independent replicates.

## Results

### MiR-574-5p is significantly highly expressed during liver fibrosis

**Fig. 1:**
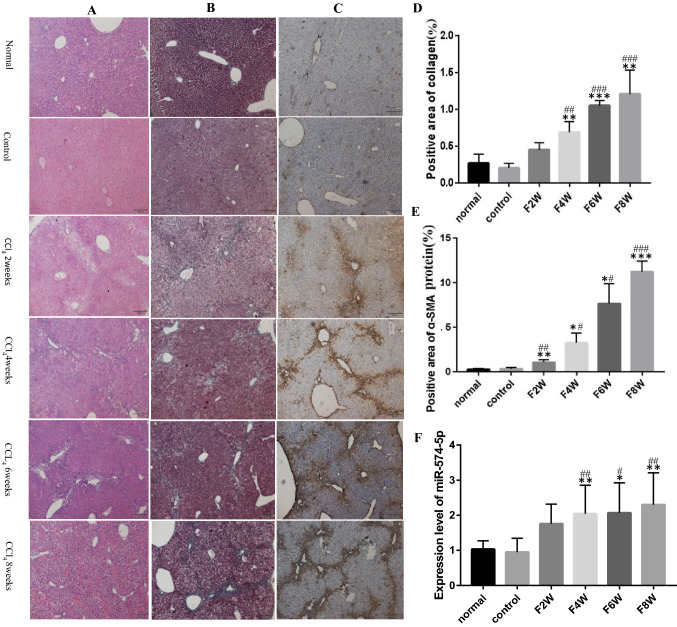
Expression of miR-574-5p in liver tissue during liver fibrosis. **A** H&E staning of live tissue. **B** Masson staining of liver tissue. Collagen deposition areas were stained blue. **C** Immunohistochemical staining of α-SMA in liver tissue. The brown granules showing positive expression of α-SMA protein were observed in liver tissue. **D** Histogram of collagen deposition area. **E** Histogram of α-SMA protein expression. **F** qRT-PCR detection of miR-574-5p in liver tissue. The column chart showed the comparison of collagen deposition and α-SMA positive area in each group, which was quantified by Image J 6.0 software. *P < 0.05, **P < 0.01, ***P < 0.001 vs normal group; #*P* < 0.05, ##*P* < 0.01, ###*P* < 0.001 vs control group.The ruler is 50 μm (black scale bar).Data represent means ± SD. (*n* = 3–6). (Color figure online)

A liver fibrosis model was established through intraperitoneal injection of 20% CCl_4_ into mice. The liver tissues of each group were subjected to H&E staining. We found that the hepatocytes of the normal group and control group were round or oval, arranged in cords around the central venous area, and distributed radially. No inflammatory cell infiltration or fiber stripe was detected in the portal area. At 2–6 weeks post modeling, there were edema, degeneration, and necrosis of hepatocytes with different degrees of inflammatory cell infiltration, particularly in the portal area. At 8 weeks post modeling, fibrous bands were connected to form fibrous septa. The remaining hepatocytes were separated into multiple pseudo lobular structures; this demonstrated the development of liver cirrhosis (Fig. 1A). Compared to the normal and control groups, Masson staining revealed that the liver of the model group showed different degrees of blue collagen deposition at 2, 4, and 6 weeks, and a larger positive area of collagen deposition; also, the positive area of collagen deposition was significantly larger at 8 weeks(*P* < 0.001) (Fig. 1B and D). Immunohistochemical results demonstrated a small amount of α-SMA was expressed in the liver tissue of normal and control groups. The positive area of α-SMA expression in the liver tissue of the model group was larger compared to that of normal and control groups at 2, 4, and 6 weeks. α-SMA expression in the liver tissue of the model group at 8 weeks was significantly higher compared to that of normal and control groups(*P* < 0.001) (Fig. 1C and E). The qRT-PCR results further confirmed that the expression of miR-574-5p in liver tissue of the model group was significantly higher at 4, 6, and 8 weeks compared to that of normal and control groups(*P* < 0.05) (Fig. 1F).

### miR-574-5p is highly expressed in human and mouse serum exosomes during liver fibrosis

**Fig. 2:**
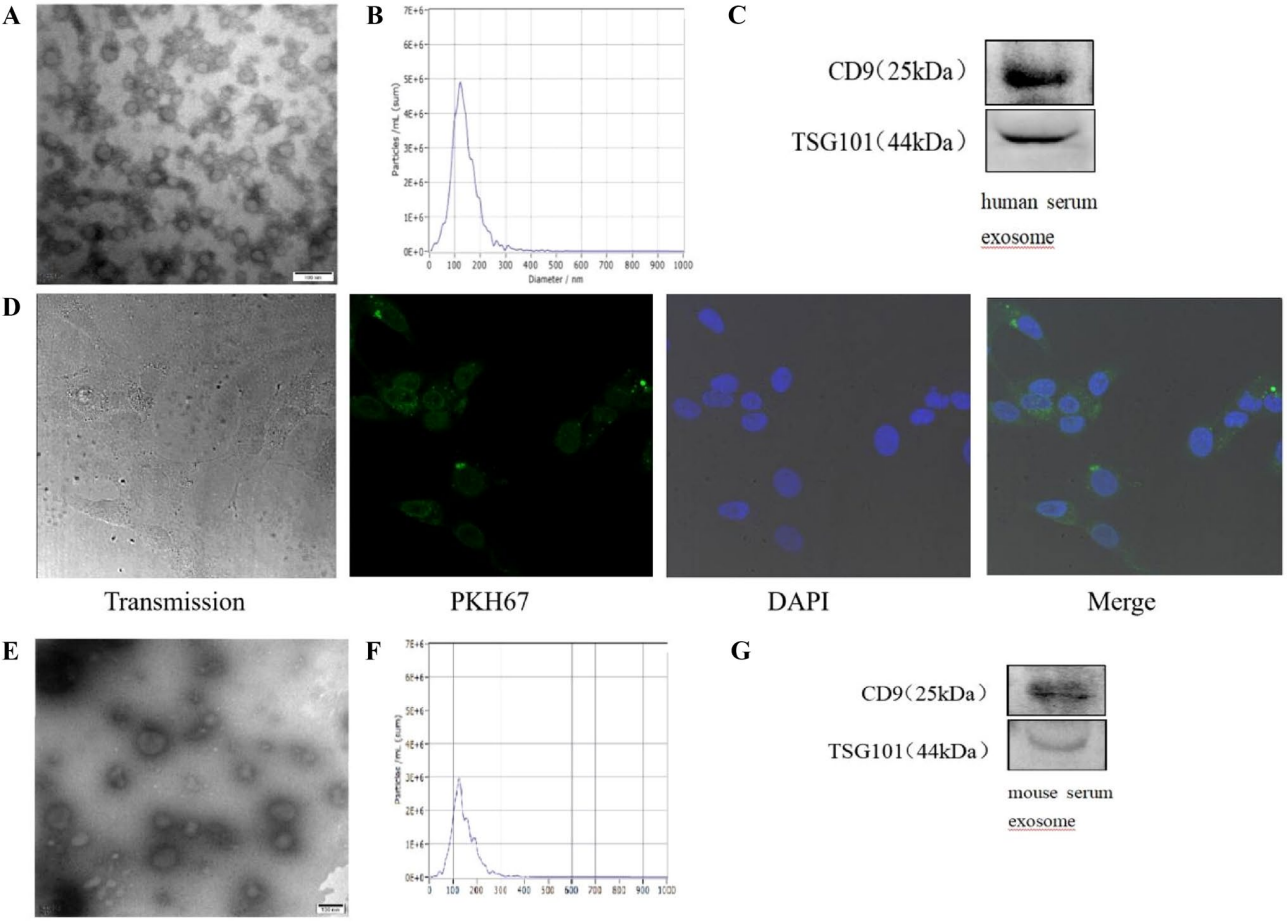
**A** The exosomes of human serum were observed by electron microscopy. **B** The size of human serum exosomes were measured by nanoparticle analysis. **C** The expression of CD9 and TSG101 of human serum exosomes were detected by Western blot. **D** Serum exosomes are internalized by LX-2. PKH:green,DAPI:blue,exosomes were dyed green. **E** The exosomes of mouse serum were observed by electron microscopy. **F** The size of mouse serum exosomes were measured by nanoparticle analysis. **G** The expression of CD9 and TSG101 of mouse serum exosomes were detected by Western blot. (Color figure online)

**Fig. 3:**
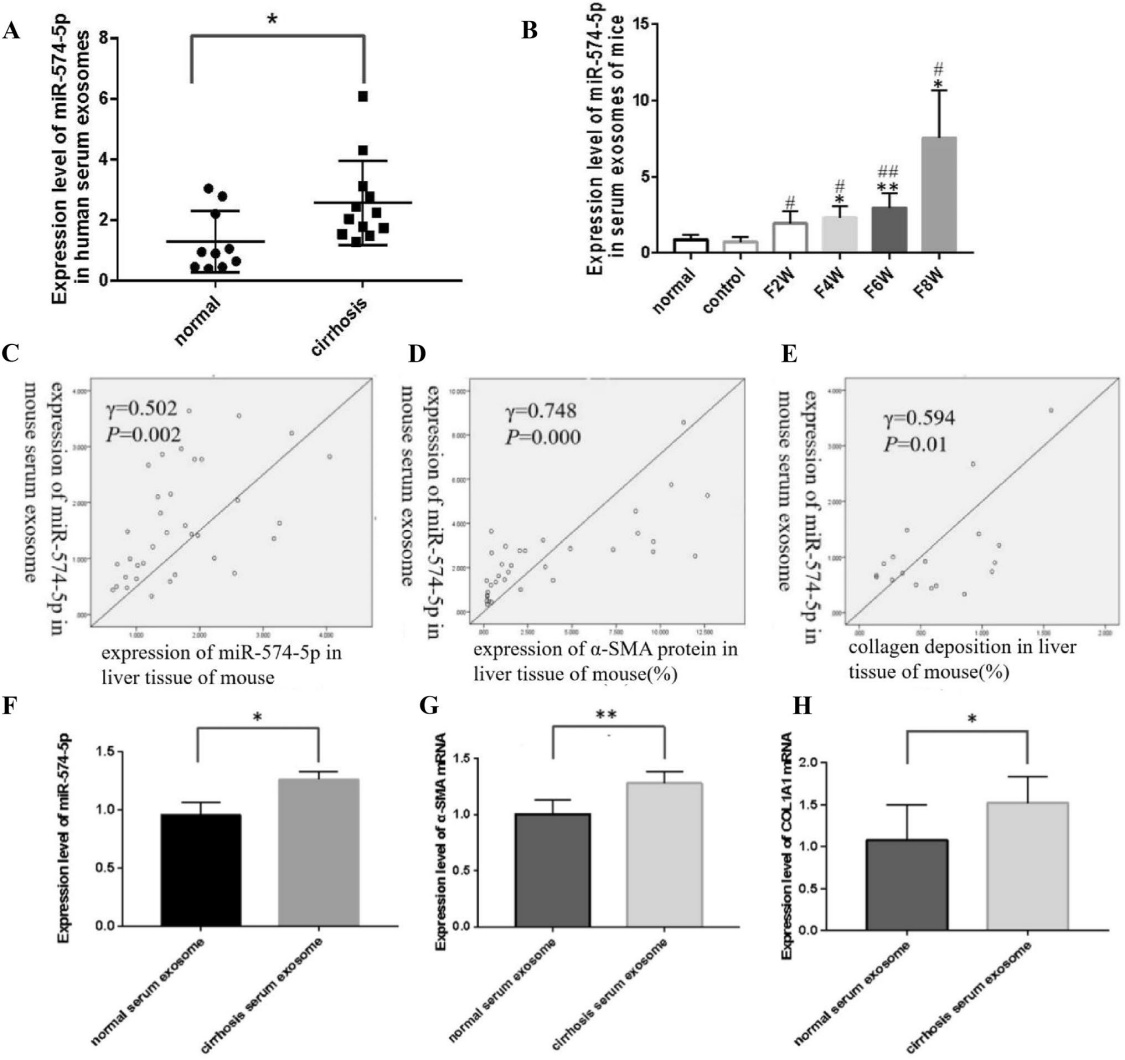
**A** Expressions of miR-574-5p in serum exosomes of patients with cirrhosis and normal healthy adults. **B** Expressions of miR-574-5p in serum exosomes of mice during liver fibrosis. **C** Correlation of mouse serum exosome miR-574-5p with exprssions of miR-574-5p in mouse liver during liver fibrosis. **D** Correlation of mouse serum exosome miR-574-5p with exprssions of α-SMA protein in mouse liver during liver fibrosis. **E** Correlation of mouse serum exosome miR-574-5p with collagen deposition in mouse liver during liver fibrosis. **F** Expression of miR-574-5p in LX-2. (*n* = 3–6). **G** Expression of α-SMA mRNA in LX-2. **H** Expression of COL1A1 mRNA in LX-2. *P < 0.05, **P < 0.01, ***P < 0.001 vs normal group; #*P* < 0.05, ##*P* < 0.01, ###*P* < 0.001 vs control group. Data represent means ± SD. (*n* ≥ 3)

Round or oval serum exosomes of human and mice were observed under an electron microscope. NTA detection revealed that the exosomes were about 120 nm in diameter. Westernblot confirmed the expression of CD9 and TSG101 (Fig. 2A–C, E–G). qRT-PCR demonstrated that the expression of miR-574-5p in serum exosomes of patients with liver cirrhosis was significantly higher than that of healthy adults (Fig. 3A). Besides, the expression of miR-574-5p in serum exosomes of mice increased gradually at 2, 4, 6, and 8 weeks during liver fibrosis, and peaked at 8 weeks. The expression of miR-574-5p in serum exosomes of model groups was significantly higher compared to normal and control groups(*P* < 0.05) (Fig. 3B). Moreover, the expression of miR-574-5p in mouse serum exosomes was positively correlated with HSC activation, collagen deposition, and the expression of miR-574-5p in liver tissues(*P* < 0.05) (Fig. 3C–E).

### Serum exosomes are internalized into HSC

We established the effect of serum exosomes on HSC whereby human serum exosomes were extracted, and co-cultured with human hepatic stellate cell line LX-2. We observed round or oval exosomes under an electron microscope. NTA detection revealed that the exosomes were about 120 nm in diameter. Westernblot confirmed the expression of CD9 and TSG101 (Fig. 2A–C). After PKH67 staining, exosomes co-cultured with LX-2 for over 12 h appeared green around the nucleus (Fig. 2D).

### Serum exosomes derived miR-574-5p potentially influence activation of hepatic stellate cells

With the development of liver fibrosis, HSC was activated. Consequently, the expression of miR-574-5p in serum exosomes and liver tissues increased. We co-cultured the serum exosomes from healthy adults and liver cirrhosis patients with HSC for 24 h respectively, and analyzed the expression of miR-574-5p, COL1A1, and α-SMA. The results showed that miR-574-5p expression in HSC co-cultured with serum exosomes of liver cirrhosis patients was higher than that of healthy adults (*P* < 0.05) (Fig. 3F). Considering the previous results, high miR-574-5p expression was associated with the increase of α-SMA and formation of liver collagen. Also, the expressions of α-SMA and COL1A1 co-cultured with serum exosomes of liver cirrhosis patients were significantly higher compared to that of healthy adults (*P* < 0.05) (Fig. 3G and H). Based on these results, we speculate that serum exosomes potentially influenced HSC activation by transferring miR-574-5p to HSC.

### MiR-574-5p overexpression promotes HSC activation and collagen synthesis

**Fig. 4:**
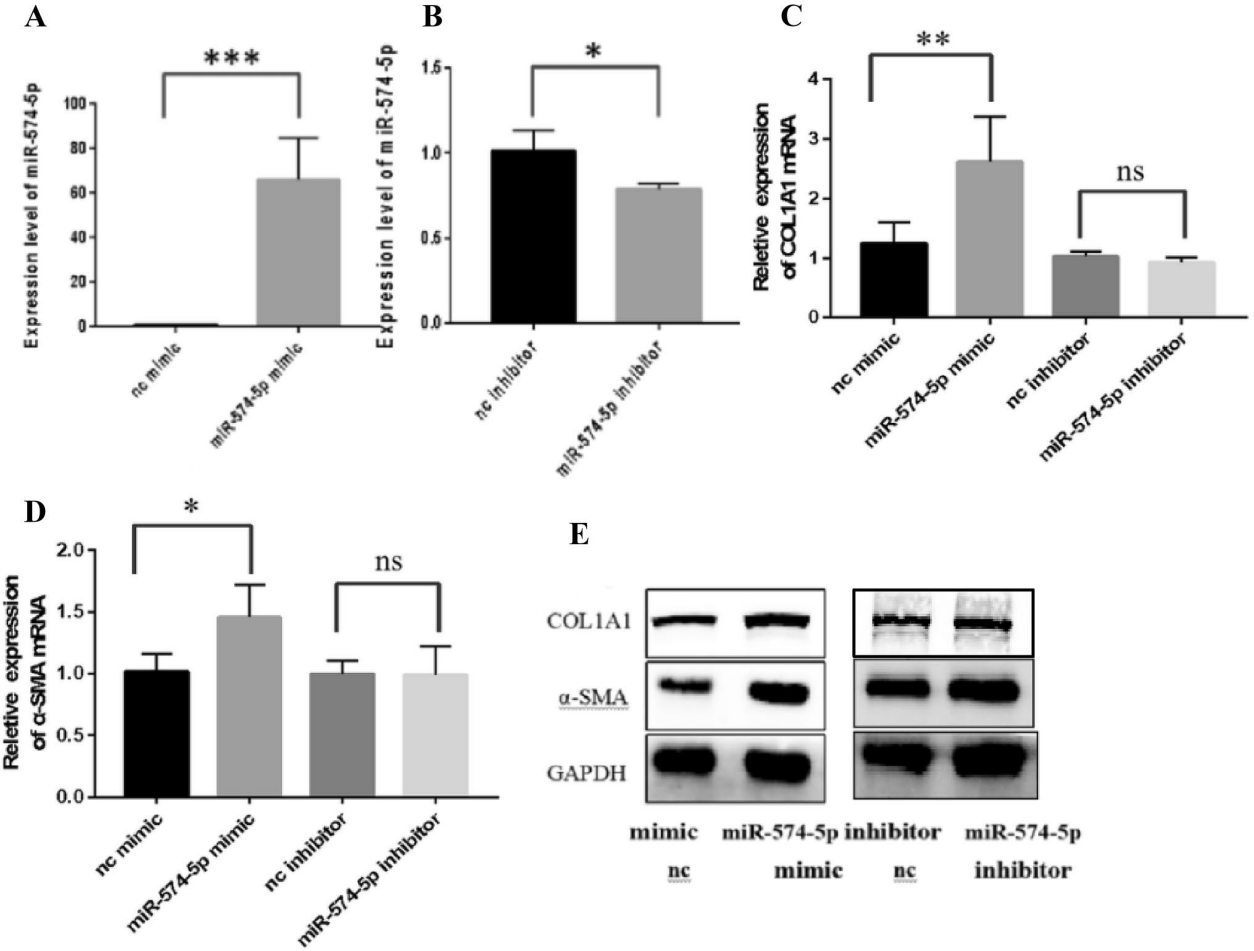
Effect of miR-574-5p on the expression of COL1A1 and α-SMA in LX-2.Expression of miR-574-5p in LX-2 transfected with **A** miR-574-5p mimic and **B** miR-574-5p inhibitor. **C** Expression of COL1A1 mRNA in LX-2 transfected with miR-574-5p mimic and miR-574-5p inhibitor. **D** Expression of α-SMA mRNA in LX-2 transfected with miR-574-5p mimic and miR-574-5p inhibitor. **E** Expression of COL1A1 and α-SMA protein in LX-2 transfected with miR-574-5p mimic and miR-574-5p inhibitor. *P < 0.05, **P < 0.01, ***P < 0.001 vs nc group. ns:no signifacance. Data represent means ± SD. (*n* ≥ 3)

To further elucidate the effect of miR-574-5p on HSCs, we transfected LX-2 with miR-574-5p mimic and miR-574-5p inhibitor, then detected the expression of COL1A1 and α-SMA. Compared to mimic NC, the expressions of COL1A1 and α-SMA mRNA and protein increased significantly in LX2 transfected with miR-574-5p mimic(*P* < 0.01) (Fig. 4C, E and G), however, miR-574-5p inhibitor had no effect on the expressions of COL1A1 and α-SMA mRNA and protein(*P* > 0.05) (Fig. 4D, F and G).

## Discussion

HSC activation is the central event in liver fibrosis [23]. Exosomes exert a crucial role in the process of HSC activation and liver fibrosis [24]. Herein, we found miR-574-5p was highly expressed in serum exosomes of mouse during liver fibrosis. The expression of miR-574-5p in serum exosomes was positively correlated with HSC activation and collagen deposition. Furthermore, we confirmed that miR-574-5p promotes HSC activation and collagen synthesis, and serum exosomes potentially activate HSC via transferring miR-574-5p to HSC during liver fibrosis.

Liver fibrosis is a common pathological process of sustained injury of liver cells caused by various chronic liver diseases. Following an injury to the liver, HSCs transform into myofibroblast-like cells and synthesize a large amount of extracellular matrix, one of the most important sections associated with liver fibrosis [25]. miRNA, a small (20-24nt) noncoding RNA, blocks mRNA expression through specific interaction with the 3’untranslated regions (UTR) of target gene mRNA [26]. Studies have revealed that various miRNAs are abnormally expressed in the process of liver fibrosis, and they drive the activation, proliferation, and apoptosis of HSCs by regulating various signaling pathways [27, 28]. Jeongeun Hyun performed a miRNA microarray analysis on liver tissues of normal and CCl_4_ treated mice and revealed that the expression of four miRNAs in the CCl_4_ treated liver was two times higher compared to the control group. In particular, the expression of miR-574-5p increased significantly [22]. We established a mouse model of liver fibrosis and detected the expressions of miR-574-5p in liver tissue at 2, 4, 6, and 8 weeks. The results showed that compared with the normal group and the control group, the expressions of miR-574-5p in the liver tissue of the model group increased at 2, 4, 6, and 8 weeks, especially at 8 weeks(*P* < 0.01). The results were consistent with the literature reports.

The exosome is a small vesicle body of about 30 ~ 150 nm in diameter and can be applied as a biological marker to monitor and evaluate the diagnosis and treatment of various liver diseases [29]. Exosomes have an outer lipid membrane and are relatively stable in serum. This explains why serum exosomes can be used for liquid biopsy in non-invasive detection and staging prediction of liver diseases [30]. Meanwhile, the surface of exosomes is rich in specific markers, and while in the serum, they can directly reflect the metabolism of the body, and be applied to evaluate disease progression, treatment, and prognosis [31]. Previous studies found that significantly high levels of miR-574-5p were expressed in liver tissue during the progression of liver fibrosis. Herein, we found that the expression of miR-574-5p in serum exosomes of patients with liver cirrhosis was significantly higher than that of healthy adults. As liver fibrosis progressed in mice, the expression of miR-574-5p in serum exosomes of model groups was significantly higher than that of normal and control groups at 4, 6, and 8 weeks. Furthermore, the expression level of miR-574-5p in serum exosomes was positively correlated with the expression of HSC activation marker α-SMA、collagen deposition and miR-574-5p in liver tissue.

Serum exosomes can be ingested by HSC. To regulate the process of liver fibrosis, exosomes mainly act as the carrier of intercellular signal transmission [13]. Studies have revealed that normal mouse serum exosomes can inhibit HSC proliferation and the expression of fibrosis-related molecules, and alleviate liver fibrosis in mice [14]. In the present study, we used PKH67 to label human serum exosomes and found that human serum exosomes were engulfed by human hepatic stellate cell line LX-2. Also, the expressions of α- SMA and COL1A1 in LX-2 co-cultured with normal serum exosomes were significantly lower than that in LX2 co-cultured with cirrhotic serum exosomes(*P* < 0.05). The expression of miR-574-5p in LX-2 co-cultured with cirrhosis serum exosomes was significantly higher compared to that in LX-2 co-cultured with normal serum exosomes. We speculated that cirrhosis serum exosomes may promote fibrosis through the transfer of miR-574-5p to LX-2.

Previous studies have shown that miR-574-5p is related to proliferation and migration of tumor cells, miR-574-5p promotes cell proliferation and migration [32, 33].Elsewhere, The effection of miR-574-5p in fibrotic diseases are rarely reported. Kenneth M.Sterling showed that two of the mouse COL3A1 first intron expressed sequence tags(EST)’s have significant identity to mmu-miR-466f-3p and mmu-miR574-5p, the predicted targets of mmu-miR-466f-3p include COL1A1、COL19A1、COL11A2、COL4A1、COL4A5, indicating that COL3A1 intronic miRNA, such as mmu-miR-574-5p may regulate the expression of collagen genes [34]. Recent years, it has been found that miR-574-5p is involved in fibroblast differentiation and collagen synthesis. Juncui found that TGF-β stimulated the expression of miR-574-5p in human cardiac fibroblasts. ARID3A is a DNA conjugation protein that negatively regulates tumor cell metastasis and invasion. MiR-574-5p promotes cardiac fibroblast proliferation and differentiation by targeting mRNA and protein levels of ARID3A [35]. However, there is no report on the role of miR-574-5p in liver fibrosis. Herein, we transfected LX-2 with miR-574-5p mimic and miR-574-5p inhibitor and found that miR-574-5p could promote the expression of α-SMA and COL1A1 in LX-2. But, inhibition of miR-574-5p exerted no effect on the expression of α-SMA and COL1A1. Our research demonstrated that miR-574-5p inhibitor had no effect on LX-2, as Jun Cui reported miR-574-5p can mediate TGF-β-induced human cardiac fibroblasts differentiation, we speculated that due to competitive inhibition, miR-574-5p couldn’t exert inhibititory effects, secondly, miR-574-5p may exert its inhibitory function under the action of TGF-β. In brief, these findings demonstrated that the expression of serum exosome miR-574-5p was significantly elevated during the progression of liver fibrosis. Consequently, serum exosome may activate HSC and promote liver fibrosis through the transfer of miR-574-5p to HSC.

## Conclusion

The present study demonstrates that the expression of serum exosome miR-574-5p is closely related to liver fibrosis. Serum exosome miR-574-5p promotes HSC activation and collagen synthesis and may be adopted as a serum marker for liver fibrosis. Thus, miR-574-5p could serve as a novel therapeutic target in liver fibrosis.

## Data Availability

The authors declare that all data supporting the conclusion of this study are available in the article.
